# Acceleration Characteristics of Discrete Fragments Generated from Explosively-Driven Cylindrical Metal Shells

**DOI:** 10.3390/ma13092066

**Published:** 2020-04-30

**Authors:** Mingxue Zhou, Cheng Wu, Fengjiang An, Shasha Liao, Xiaoxia Yuan, Dongyu Xue, Jian Liu

**Affiliations:** State Key Laboratory of Explosion Science and Technology, Beijing Institute of Technology, Beijing 100081, China; zmxbit@163.com (M.Z.); anfengjiang@bit.edu.cn (F.A.); haha121@bit.edu.cn (S.L.); 3120160144@bit.edu.cn (X.Y.); xuedychn@gmail.com (D.X.); 2120170237@bit.edu.cn (J.L.)

**Keywords:** shell disintegration, gas leakage, discrete fragments, acceleration

## Abstract

The acceleration characteristics of fragments generated from explosively-driven cylindrical shells are important issues in warhead design. However, there is as yet no reasonable theory for predicting the acceleration process of a specific metallic shell; existing approaches either ignore the effects of shell disintegration and the subsequent gas leakage on fragment acceleration or treat them in a simplified manner. In this paper, a theoretical model was established to study the acceleration of discrete fragments under the combined effect of shell disintegration and gas leakage. Firstly, an equation of motion was developed, where the acceleration of a cylindrical shell and the internal detonation gas was determined by the motive force impacting the inner surface of the metallic cylinder. To account for the force decrease induced by both the change in fragment area after the shell disintegrates and the subsequent drop in gas pressure due to gas leakage, the equation of motion was then associated with an equation for the locally isentropic expansion of the detonation gas and a modified gas-leakage equation. Finally, theoretical analysis was conducted by solving the associated differential equations. The proposed model showed good agreement with experimental data and numerical simulations, indicating that it was suitable for predicting the acceleration of discrete fragments generated from a disintegrated warhead shell. In addition, this study facilitated a better understanding of the complicated interaction between fragment acceleration and gas outflow.

## 1. Introduction

Cylindrical shells filled with explosive charges are typical structures in conventional warhead design and can be sorted into the continuous shell and the preformed-fragment shell. After detonation, the metallic shells are driven to accelerate rapidly by the high-intense shock and internal detonation products, followed by large plastic deformations that ultimately lead to disintegration [1,2,3,4]. The acceleration characteristics and final velocity of fragments generated from the disintegrated shells have been of interest to the researchers for decades in the field of the structure protection and weapon effectiveness. 

In 1943, Gurney [5] proposed a formula for the initial velocity of fragments generated from a cylindrical metal shell filled internally with explosives, namely:

(1) $$v_{0}=\sqrt{2E}\sqrt{\frac{\beta }{1+0.5\beta }}$$

where $\sqrt{2E}$ is the Gurney energy of the explosive, $v_{0}$ is the initial velocity of fragments, and *β* is the ratio of the charge mass to that of the metal shell. The Gurney formula laid the foundation for subsequent research on final fragment velocity and has been widely cited and adapted [6,7,8,9]. Several studies [10,11] have shown that for a copper shell, the Gurney formula fits well with the maximum fragment velocity. However, the Gurney formula ignores the shell disintegration history and the detonation gas leakage process, during which the energy dissipates through fragments gaps. Thus, for metal shells that are either less ductile than copper or disintegrate prematurely, the final fragment velocity is inevitably lower than that predicted by the Gurney formula [12,13,14,15].

In an effort to find a method for predicting the acceleration history of an explosively-driven shell, Koch [16] derived the following formula based on energetic considerations:

(2) $$V(t)=\sqrt{\frac{2}{\gamma -1}\frac{P_{0}}{\rho_{0}}/\left(\frac{n}{n+2}+\frac{M}{C}\right)}{\left[1-{\left(\frac{R_{0}}{R\left(t\right)}\right)}^{n\left(\gamma -1\right)}\right]}^{1/2}$$

where *V(t)* is the expanding velocity of the shell, *R*_0_ is the initial inner radius of the shell, and *R(t)* is the expanding radius of the shell. *γ* is the polytrophic exponent of the expanding gas. $\rho_{0}$ and $P_{0}$ are the initial density and pressure of the homogeneous gas products, respectively. *n* is the geometry parameter of the structure, with *n =* 2 corresponding to a cylindrical system. The first term on the right-hand side of Equation (2) corresponds exactly to Gurney’s expression for the fragment velocity in the derivation [16]. Thus, Equation (2) can be rewritten as [17,18]

(3) $$V(t)=V_{G}{\left[1-{\left(\frac{R_{0}}{R\left(t\right)}\right)}^{n\left(\gamma -1\right)}\right]}^{1/2}$$

where $V_{G}$ is the fragment velocity predicted by the Gurney formula. Lindsay et al. [19] and Elek et al. [20] noted that a cylindrical shell was driven simultaneously by the shock wave and the detonation product in the initial stage of shell expansion. Lindsay et al. [19] separated the roles of the two factors and established a two-component model to fit the shell expansion data obtained from a copper-cylinder expansion test. However, their formulas neglect shell disintegration and gas leakage, and consequently the post-disintegration acceleration of the shell is again not derived or described reasonably.

Considering the breaking up effect of a continuous cylindrical shell, Taylor [21] firstly established a model of the radial-fracture process of the shell by introducing the strength and yield criteria. However, Taylor’s radial-fracture theory is only applicable to the shells with either a low strain rate or a thick wall. Hoggatt and Recht [22] then perfected Taylor’s theory by the mechanism of shear fracture. Zhang and Sun [23] built a model for predicting the fracture radius of a cylindrical shell and the corresponding rupture velocity, based on yield conditions, continuous equations, and equations of motion. These models have contributed successfully to the understanding of the failure and fracture behavior of continuous warhead shells, but unfortunately it remains challenging to calculate the specific fragment acceleration process because gas leakage is much too complicated and challenging to analyze.

Recently, Hutchinson et al. [18] built a model to describe the gas leakage and the fragment acceleration in the post-fracture process of a continuous cylindrical shell. The theory describes clearly the escape of gas through fragment gaps after a warhead shell breaks up. However, the fragment acceleration is considered to be independent of the gas leakage in the calculation. In addition, the model is based on the assumption of the isentropic expansion (also treated as adiabatic expansion) of the detonation gas inside an expanding shell, which is essentially non-isentropic after the shell loses continuity because the leaked gas product causes a drop in gas pressure and heat dissipates into the external atmosphere. As a result, the model proposed by Hutchinson requires some modification. Charron [24] proposed a revised Gurney formula to calculate the velocities of preformed fragments, replacing *β* (the ratio of charge mass to shell mass) with 0.8*β* to account for the effect of gas leakage. Similarly, Kim et al. [25] reduced the effective charge mass to calculate the fragment velocity. The correction coefficients were found to depend on the fragment shape rather than their size or material. However, the calculation of gas leakage is not based on a strict derivation; both aforementioned simple modifications of the Gurney formula are based on fits of experimental data or numerical simulation, and the correction coefficients might vary with the shell structure and the explosive type.

Note that the application of Photonic Doppler Velocimetry (PDV) arrays and high-speed framing cameras has provided valuable experimental data on the shell acceleration and disintegration [3,4,26,27]. However, to date the exact mechanism for the post-disintegration acceleration of a warhead shell remains to be discovered, this being because of the complicated interaction between fragment acceleration and gas outflow. In order to facilitate the warhead design, it is necessary to predict the whole acceleration characteristics of a warhead shell accurately. In the present work, a theoretical model was established to account for the combined effect of shell disintegration and gas leakage on fragment acceleration. The detailed fragment-acceleration process and final fragment velocities agreed well with experimental data and numerical simulations, indicating that the proposed model is applicable to calculate the acceleration of discrete fragments produced by a warhead shell.

## 2. Theoretical Analysis

The motive force, namely the product of detonation gas pressure and the inner surface area of a cylindrical shell, is assumed to push the shell and internal detonation gas outwards. Before the shell disintegrates, the internal gas pressure decreases continuously as the shell expands outwards. However, after the shell fractures or separates into discrete fragments, the decrease in gas pressure is induced by both the expansion and gas escaping through fragments gaps. In addition, the inner surface area of the shell ceases to increase after the cylinder loses continuity. Therefore, the motive force and motion characteristics of the disintegrated shell differ greatly from those of the non-disintegrated shell, so we divide the shell acceleration process into two stages, namely pre-disintegration and post-disintegration.

### 2.1. Equation of Motion

**Figure 1:**
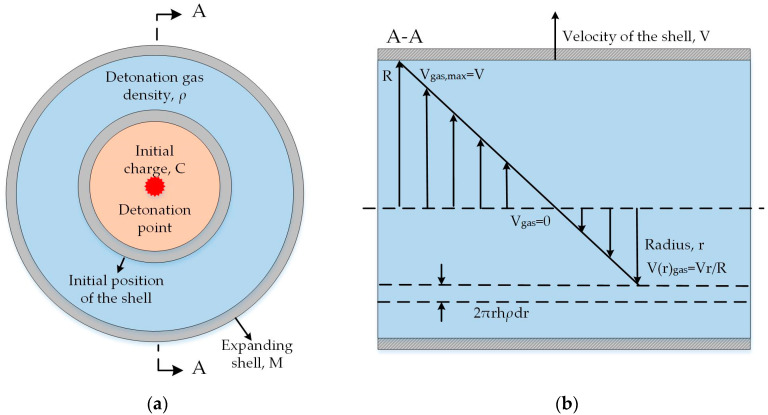
Cylindrical geometry of expanding shell driven by detonation gas: (**a**) vertical view of the shell; (**b**) cross-sectional view of the shell.

An equation of motion for a continuous cylindrical metallic shell and its internal detonation gas was first established. The geometry of the cylindrical shell is shown in Figure 1. We introduce the following assumptions to simplify the analysis and derivation process.

1. The explosive detonates instantaneously, after which the detonation gas inside the shell has a uniform density of *ρ*. The velocity $V{\left(r\right)}_{gas}$ of the detonation gas increases linearly from the charge center to the inner surface of the shell, as shown in Figure 1b. Then, for any radial position *r* inside the shell, we have
  (4) $$V(r){}_{gas}=V\cdot r/R$$
  where *V* is the velocity of the inner surface of the shell, and *R* is the expanding inner radius of the shell.
2. The shell fractures radially. After the shell ruptures, fragments scatter at the same radial velocity with a fixed orientation. The mass loss of the fragments during the explosion is neglected.
3. The cylindrical shell is assumed to be infinite. We investigate the motion of the detonation gas and the shell cut out from the central part of the shell, where the end effect induced by the rarefaction wave can be neglected. Thus, the problem can be treated as a two-dimensional (2D) problem.

The equation of motion for the detonation gas and the cylindrical shell can be expressed as

(5) $$F=SP=2\pi RhP=M\frac{dV}{dt}+\int_{0}^{R}2\pi rh\rho \frac{dV{\left(r\right)}_{gas}}{dt}dr=(M+\frac{2C}{3})\frac{dV}{dt}=(M+\frac{2C}{3})V\frac{dV}{dR}$$

where *F*, *S*, and *P* are the motive force acting on the detonation gas and the shell, the internal area of the shell, and the gas pressure inside the shell, respectively. *M* is the mass of the shell, *C* is the mass of the initial charge, and *V* is the velocity of the inner surface of the shell. *R* is the expanding internal radius of the shell, and *h* is the unit length of the shell. From the right-hand side of Equation (5), the mass of detonation gas that accelerates synchronously with the shell is 2*C*/3. Thus, the surface area of detonation gas on which the motive force acts can be regarded as that of the warhead shell. 

### 2.2. Pre-Disintegration Acceleration

Taking $R_{0}$ as the initial inner radius of the shell, we obtain

(6) $$\frac{S}{S_{0}}=\frac{2\pi Rh}{2\pi R_{0}h}=\frac{R}{R_{0}}$$

For the isentropic or adiabatic expanding gas, the gas pressure *P* and density *ρ* can be expressed as [18]

(7) $$\frac{P}{P_{0}}={\left(\frac{\rho }{\rho_{0}}\right)}^{\gamma }={\left(\frac{R_{0}}{R}\right)}^{2\gamma }$$

where $P_{0}$ and $\rho_{0}$ are the initial detonation pressure and density of the charge, respectively. *γ* is the polytropic exponent of the isentropic expansion, with *γ* = 3 being a good estimation for most explosives.

From shock theory, it is known that

(8) $$P_{0}=\frac{\rho_{0}}{\gamma +1}{\left(\frac{\gamma }{\gamma +1}\right)}^{\gamma }D^{2}$$

where *D* is the detonation velocity of the explosive [16]. Then Equation (7) can be transformed into

(9) $$P=\frac{\rho_{0}D^{2}}{\gamma +1}{\left(\frac{\gamma }{\gamma +1}\right)}^{\gamma }{\left(\frac{R_{0}}{R}\right)}^{2\gamma }$$

Substituting Equations (6) and (9) into Equation (5), yields

(10) $$\frac{3+2\beta }{3\beta }VdV=\frac{2D^{2}}{\gamma +1}{\left(\frac{\gamma }{\gamma +1}\right)}^{\gamma }\frac{R_{0}^{2\gamma -2}}{R^{2\gamma -1}}dR$$

where *β = C/M*. Integrating Equation (10) gives the acceleration equation of the shell before disintegration, namely

(11) $$V(R)=\sqrt{\frac{2D^{2}}{\gamma^{2}-1}{\left(\frac{\gamma }{\gamma +1}\right)}^{\gamma }\frac{3\beta }{3+2\beta }}\cdot \sqrt{\left[1-{\left(\frac{R_{0}}{R}\right)}^{2\gamma -2}\right]}$$

### 2.3. Post-Disintegration Acceleration

After the shell ruptures into discrete fragments, Equation (5) can be transformed into

(12) $$F{}^{\prime }=S{}^{\prime }\cdot P{}^{\prime }=2\pi R_{f}h\cdot P{}^{\prime }=\left[M+\frac{2}{3}C(1-\frac{C{}^{\prime }}{C})\right]V\frac{dV}{dR}$$

where $F{}^{\prime }$, $S{}^{\prime }$, and $P{}^{\prime }$ are the motive force, the inner surface area of the disintegrated shell, and the gas pressure inside the disintegrated shell, respectively. $R_{f}$ is the fracture radius of the shell, and $C{}^{\prime }$ is the gaseous mass loss, where $C{}^{\prime }/C$ represents the mass ratio of leaked gas to total charge. The problem addressed in Section 2.3.1 and Section 2.3.2 is how to derive expressions for $P{}^{\prime }$ and $C{}^{\prime }/C$.

#### 2.3.1. Equation for Locally Isentropic Expansion

Before a warhead shell breaks up, the internal detonation gas is isolated from the outside atmosphere without heat exchange, so the gas expansion inside the shell can be treated as isentropic. However, after the shell ruptures or separates into discrete fragments, the detonation gas leaks out, thereby decreasing the internal detonation gas pressure. Meanwhile, the heat dissipation occurs, so the gas expansion can no longer be considered as isentropic. Fortunately, the gas expansion inside the disintegrated shell can be regarded as locally isentropic. That is, the gas expansion process can be divided into many infinitesimal sections, during which gas leakage and heat dissipation are negligible, thereby allowing the gas expansion to be regarded as locally isentropic.

**Figure 2:**
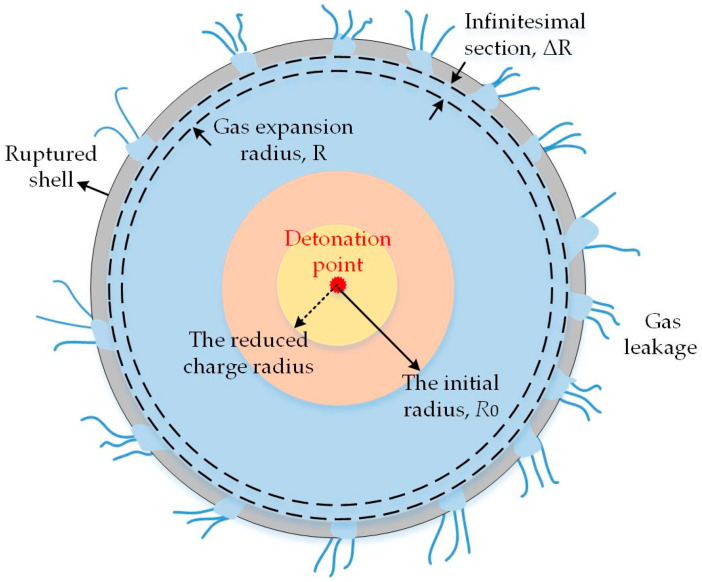
Illustration of locally isentropic expansion of detonation gas after shell ruptures.

Before the expanding radius *R*, gas has escaped through fragment gaps and decreased the gas pressure *P* and density *ρ* inside the ruptured shell compared to their values inside the non-ruptured shell. The initial charge is assumed to be reduced to account for the decline in gas pressure, density, and mass induced by gas leakage. In other words, the mass of the effective initial charge corresponds to that of the remaining gas at the expansion radius *R*. Subsequently, the gas produced by the reduced initial charge expands isentropically in the section *∆R*. This reduction in the effective charge mass is similar to the modification made by Charron [24] and Kim et al. [25]. However, the local isentropic equation is connected to the gas leakage equation and the shell acceleration equation for iterative calculations, where the gas leakage, gas pressure decline, and fragment acceleration are all connected. The locally isentropic expansion of detonation gas is depicted in Figure 2.

Then, Equation (7) can be transformed into

(13) $$\frac{P{}^{\prime }}{P_{0}}={\left(\frac{\rho {}^{\prime }}{\rho_{0}}\right)}^{\gamma }={\left(\frac{R_{0}^{\prime }}{R}\right)}^{2\gamma }={\left(\frac{R_{0}}{R}\cdot \frac{R_{0}^{\prime }}{R_{0}}\right)}^{2\gamma }={\left(\frac{R_{0}}{R}\right)}^{2\gamma }{\left(\frac{C_{remain}}{C}\right)}^{\gamma }={\left(\frac{R_{0}}{R}\right)}^{2\gamma }{\left[1-\frac{C{}^{\prime }}{C}\right]}^{\gamma }$$

where $P{}^{\prime }$ and $\rho {}^{\prime }$ are the gas pressure and density inside the ruptured shell during the locally isentropic expansion section *∆R*, respectively. $R_{0}^{\prime }$ is the reduced initial radius of charge, whose mass is the remaining gas mass $C_{remain}$.

Substituting Equation (8) into Equation (13), yields

(14) $$P{}^{\prime }=\frac{\rho_{0}D^{2}}{\gamma +1}{\left(\frac{\gamma }{\gamma +1}\right)}^{\gamma }{\left(\frac{R_{0}}{R}\right)}^{2\gamma }{\left(1-\frac{C{}^{\prime }}{C}\right)}^{\gamma }$$

which is an expression for $P{}^{\prime }$ considering the effect of gas leakage. A corresponding expression for $C{}^{\prime }/C$ is presented in Section 2.3.

#### 2.3.2. Model of Gas Leakage

As mentioned previously, the gas leakage theory from Hutchinson et al. [18] is based on the assumption of isentropic expansion of the gas inside the ruptured shells, which is actually non-isentropic because of gas outflow. In addition, the acceleration of discrete fragments is considered to be independent of gas leakage. Thus, we modified the theory somewhat, replacing isentropic detonation gas expansion with locally isentropic expansion and allowing the fragment acceleration to interact with the gas leakage process. 

After a warhead shell breaks up, the expanding gas inside the shell is assumed to escape through hypothetical “stream-tubes” in which the flow is considered as one-dimensional supersonic flow and is described by an energy-per-unit-mass conservation equation [18]

(15) $$\frac{P}{\rho }-\frac{P_{c}}{\rho_{c}}=\left(\frac{\gamma -1}{2\gamma }\right)u_{c}{}^{2}$$

where $P_{c}$, $\rho_{c}$, and $u_{c}$ denote the gas pressure, density, and outflow velocity at the crack, respectively. As mentioned in Section 2.2, the gas expansion inside the ruptured shell can be regarded as locally isentropic. Thus, Equation (15) can be transformed into

(16) $$\frac{P{}^{\prime }}{\rho {}^{\prime }}-\frac{P_{c}}{\rho_{c}}=\left(\frac{\gamma -1}{2\gamma }\right)u_{c}{}^{2}$$

Defining $P_{c}=qP{}^{\prime }$, where q<1, from Equation (13) we have

(17) $$\rho_{c}=q^{1/\gamma }\rho {}^{\prime }$$

Substituting Equation (17) into Equation (15) gives

(18) $$\frac{P{}^{\prime }}{\rho {}^{\prime }}-\frac{qP{}^{\prime }}{q^{1/\gamma }\rho {}^{\prime }}=\frac{\gamma -1}{2\gamma }u_{c}^{2}=\frac{P{}^{\prime }}{\rho {}^{\prime }}\left(1-q^{1-1/\gamma }\right)$$

Solving this quadratic equation and taking the positive root gives

(19) $$u_{c}=\sqrt{\frac{2\gamma P{}^{\prime }}{(\gamma -1)\rho {}^{\prime }}\left(1-q^{(\gamma -1)/\gamma }\right)}$$

Combining Equations (17) and (19) gives

(20) $$u_{c}\rho_{c}=q^{1/\gamma }\sqrt{\frac{2\gamma P{}^{\prime }\rho {}^{\prime }}{\left(\gamma -1\right)}}\sqrt{\left(1-q^{(\gamma -1)/\gamma }\right)}$$

Differentiating Equation (20) and taking the maximum value of $u_{c}\rho_{c}$, leads to a value for q

(21) $$q={\left(\frac{2}{\gamma +1}\right)}^{\frac{\gamma }{\gamma -1}}$$

Substituting Equation (21) into Equation (20) gives

(22) $$u_{c}\rho_{c}=\sqrt{\gamma P{}^{\prime }\rho {}^{\prime }}{\left(\frac{2}{\gamma +1}\right)}^{\frac{\gamma +1}{2(\gamma -1)}}$$

We assume that the total crack width on the warhead shell is *W*. Then *W* can be expressed as *W* = 2*π*(*R* − *R_f_*), and the initial charge mass per unit length is $C=\pi \rho_{0}R_{0}^{2}h$. From Equation (13), the gas pressure $P{}^{\prime }$ and density $\rho {}^{\prime }$ at radius *R* can be expressed in terms of initial detonation pressure $P_{0}$ and density $\rho_{0}$ at the initial radius $R_{0}$. Therefore, the ratio of the gas mass leaking per second from the total cracks at the fracture radius $R_{f}$ to its total mass can be written as

(23) $$\frac{u_{c}\rho_{c}W}{C}=\frac{2\left(R-R_{f}\right)}{R_{0}}\sqrt{\frac{\gamma P_{0}}{\rho_{0}R_{0}^{2}}}{\left(\frac{R_{0}}{R}\right)}^{\gamma +1}{\left(\frac{2}{\gamma +1}\right)}^{\frac{\gamma +1}{2\left(\gamma -1\right)}}{\left[1-\frac{C{}^{\prime }}{C}\right]}^{\frac{\gamma +1}{2}}$$

and the Hutchinson gas-leakage equation can be expressed as [18]

(24) $$\frac{u_{c}\rho_{c}W}{C}=\frac{2\left(R-R_{f}\right)}{R_{0}}\sqrt{\frac{\gamma P_{0}}{\rho_{0}R_{0}^{2}}}{\left(\frac{R_{0}}{R}\right)}^{\gamma +1}{\left(\frac{2}{\gamma +1}\right)}^{\frac{\gamma +1}{2\left(\gamma -1\right)}}$$

Clearly, Equation (23) contains the effect of locally isentropic expansion. Note that $C{}^{\prime }/C$ can be regarded as the ratio of leaked gas mass to the total charge mass through the total cracks *W* on the two-dimensional the warhead ring, which is equal to the integral of Equation (23), namely

(25) $$\begin{array}{ll} \frac{C{}^{\prime }}{C} & =\int_{t_{f}}^{t}\frac{2\left(R-R_{f}\right)}{R_{0}}\sqrt{\frac{\gamma P_{0}}{\rho_{0}R_{0}^{2}}}{\left(\frac{R_{0}}{R}\right)}^{\gamma +1}{\left(\frac{2}{\gamma +1}\right)}^{\frac{\gamma +1}{2\left(\gamma -1\right)}}{\left[1-\frac{C{}^{\prime }}{C}\right]}^{\frac{\gamma +1}{2}}dt\\ & =\int_{R_{f}}^{R}\frac{2\left(R-R_{f}\right)}{R_{0}}\sqrt{\frac{\gamma P_{0}}{\rho_{0}R_{0}^{2}}}{\left(\frac{R_{0}}{R}\right)}^{\gamma +1}{\left(\frac{2}{\gamma +1}\right)}^{\frac{\gamma +1}{2\left(\gamma -1\right)}}\frac{1}{V}{\left[1-\frac{C{}^{\prime }}{C}\right]}^{\frac{\gamma +1}{2}}dR \end{array}$$

where $t_{f}$ is the fracture moment of the shell, and t corresponds to the moment when the ruptured shell expands to the radius of *R*. Clearly, $C{}^{\prime }/C$ is an implicit function, the differential of which can be expressed as

(26) $$\frac{d(C{}^{\prime }/C)}{dR}=\frac{2\left(R-R_{f}\right)}{R_{0}}\sqrt{\frac{\gamma P_{0}}{\rho_{0}R_{0}^{2}}}{\left(\frac{R_{0}}{R}\right)}^{\gamma +1}{\left(\frac{2}{\gamma +1}\right)}^{\frac{\gamma +1}{2\left(\gamma -1\right)}}\frac{1}{V}{\left[1-\frac{C{}^{\prime }}{C}\right]}^{\frac{\gamma +1}{2}}$$

A closer inspection of Equations (12), (14), and (26) reveals that the leaked detonation gas will interact with the shell expansion. Initially after the shell breaks up, the gas leakage increases as the cracks extend (see Equation (23)), resulting in decreased gas pressure and motive force inside the shell compared with those in the non-ruptured shell. This critical drop hinders the fragment acceleration and causes a lower fragment velocity. However, as the warhead shell expands, the increased fragment velocity reduces the gas leakage rate (see Equation (26)), and the internal gas pressure is insufficient to maintain a high leakage rate due to expansion. As a result, the gas leakage stops when the gas outflow keeps pace with the expansion of the ruptured shell. It is difficult to derive an analytical solution for the above relationship, but results can be obtained by solving the following coupled differential equations:

(27) $$\{\begin{array}{l} 2\pi R_{f}h\cdot P{}^{\prime }=\left[M+\frac{2}{3}C(1-\frac{C{}^{\prime }}{C})\right]V\frac{dV}{dR}\\ \frac{d(C{}^{\prime }/C)}{dR}=\frac{2\left(R-R_{f}\right)}{R_{0}}\sqrt{\frac{\gamma P_{0}}{\rho_{0}R_{0}^{2}}}{\left(\frac{R_{0}}{R}\right)}^{\gamma +1}{\left(\frac{2}{\gamma +1}\right)}^{\frac{\gamma +1}{2\left(\gamma -1\right)}}\frac{1}{V}{\left[1-\frac{C{}^{\prime }}{C}\right]}^{\frac{\gamma +1}{2}}\\ P{}^{\prime }=\frac{\rho_{0}D^{2}}{\gamma +1}{\left(\frac{\gamma }{\gamma +1}\right)}^{\gamma }{\left(\frac{R_{0}}{R}\right)}^{2\gamma }{\left(1-\frac{C{}^{\prime }}{C}\right)}^{\gamma } \end{array}$$

Substituting γ=3 into Equation (27) and simplifying, yields

(28) $$\left\{ \begin{array}{l} V\frac{dV}{dR}\left[\frac{3+2\beta \left(1-C{}^{\prime }/C\right)}{3\beta }\right]=\frac{27R_{f}D^{2}}{128}\frac{R_{0}{}^{4}}{R^{6}}\cdot {\left(1-\frac{C{}^{\prime }}{C}\right)}^{3}\\ \frac{d(C{}^{\prime }/C)}{dR}=\frac{9\left(R-R_{f}\right)D}{16R_{0}{}^{2}}{\left(\frac{R_{0}}{R}\right)}^{4}\frac{1}{V}{\left[1-\frac{C{}^{\prime }}{C}\right]}^{2} \end{array}\right.$$

which determine completely the gas outflow and acceleration of discrete fragments produced by a ruptured shell. 

Solving for the acceleration of discrete fragments requires a determined fracture radius, and the established model seems more suitable for a naturally fragmenting cylinder (also known as a kind of continuous cylinder and used widely in artillery projectiles) rather than a shell with preformed fragments because the latter involves no fragmentation process. A cylindrical shell comprising preformed fragments contains initial fragment gaps through which the gas product should emerge once the cylinder starts to expand. However, previous studies [24,28] have shown that in the initial stage of preformed-fragment acceleration, the circumferential fragment gaps close as the compression wave propagates through the fragments, and the gas does not leak out until the fragments separate as a reflection wave from the free surface propagates back. As a result, a preformed-fragment cylinder can also be considered as a continuous cylinder. Herein, we use experimental data to determine the fracture radius at which the gaps between preformed fragments reopen and gas leaks out. 

Note that we treated the acceleration of warhead shell as a two-dimensional (2D) problem, but the law of gas leakage was based on a single crack through which the flow was considered as one-dimensional (see Equation (22)). It seems that the chosen approach may contradict the two-dimensional theoretical framework. However, we then assumed that the total crack width on the warhead ring was *W,* which can be expressed as *W* = 2*π*(*R* − *R_f_*). After that, we obtained the ratio of the gas mass leaking per second from the total crack *W* at the fracture radius *R_f_* to its total mass (see Equation (23)). Finally, we used the integral of Equation (23) to obtain the gas mass that leaked through the total crack *W* on the two-dimensional the warhead ring (see Equation (25)). As a result, the chosen approach can also be regarded as a two-dimensional problem. The method of dealing with gas leakage is consistent with the work of Karrp and Predebon [1] who calculated the rate of efflux from an ideal nozzle passing its maximum rate of flow. Then the gas leakage model was presented as an integral part of the code to calculate the fragments acceleration and projection angles of fragments. Comparisons were given between the code calculations with the gas leakage model and experimental data of cylindrical shells and 105 mm HE projectiles, and very good agreement was obtained.

In addition, it is possible that the size or the number of fragmentation has limited effects on the acceleration of discrete fragments. There are several possible explanations for this point of view. It can be inferred from Equation (12) and Equation (14) that the acceleration of the discrete fragments generated from the disintegrated shell is determined by the inner surface of the fractured shell $S{}^{\prime }$, the gas pressure inside the disintegrated shell $P{}^{\prime }$, and the ratio of the leaked charge mass to the total mass $C{}^{\prime }/C$. Firstly, regardless of the degree or the size of fragmentation, the internal surface area of the fractured shell remains constant. That is to say, the inner surface of the fractured shell is only related to the fracture radius *R_f_* and not to the size of the fragmentation. Secondly, $P{}^{\prime }$ is determined by $C{}^{\prime }/C$. Thirdly, it can be inferred from Equations (23) and (25) that the gas leakage is still related to the fracture radius *R_f_* and not to the size of the fragmentation. Taken together, these results may suggest that the acceleration of discrete fragments is likely to be determined by the fracture radius *R_f_*.

## 3. Verification of Theoretical Model

In this section, we present theoretical calculations to study the accelerations of a naturally fragmenting cylinder and a preformed-fragment one, and we use experimental data and simulation results to verify the proposed model. A code in Matlab software (R2014b, MathWorks, Natick, MA, USA) was used to calculate the detailed fragment acceleration and the gas leakage by solving Equations (11) and (28), where all the variables are calculated iteratively at each 1% step in expansion ratio of $R/R_{0}$.

### 3.1. Natural Fragment Acceleration

For our theoretical calculations on natural fragment acceleration, we choose to replicate the experiment conducted by Wang et al. [4], because the shell disintegration and acceleration process of an AISI 1045 steel cylinder were captured clearly and the place where PDV located for fragment velocity measurement was free from end effects. The critical parameter values for calculation are *R*_0_ = 25 mm, *β* = 0.40, *γ* = 3, *D* = 6700 m/s, *R_f_* = 45.75 mm, *V_f_* = 1167 m/s, as shown in Table 1.

**Figure 3:**
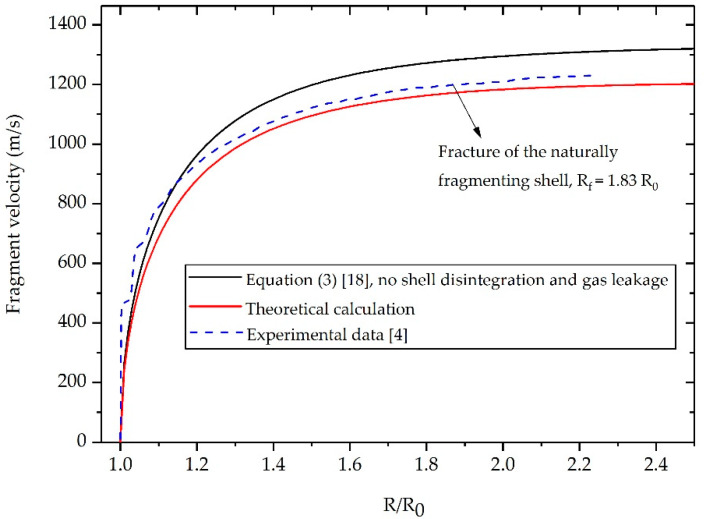
Velocity histories of fragments generated from a naturally fragmenting shell made from AISI 1045 steel.

The fragment velocity predicted by the theoretical model along with the data from Wang’s experiment [4] and results calculated by Equation (3) are plotted in Figure 3. We divide the velocity curves into three stages according to the motion features of the cylindrical shells. The first stage is between the start of shell expansion by detonation and the disappearance of oscillation in experimental data. In this stage, the cylindrical shells expand rapidly under the initial high-pressure detonation front. As shown in Figure 3, the experimental data for fragment velocity is slightly higher than the theoretical calculation. This can be explained by the fact that the shell in the experiment is accelerated by the high-pressure detonation front initialing from the charge center. The detonation wave propagates from the center of charge to the interface between the charge and the shell, during which the detonation front continues to increase. However, in this article, the detonation of explosives is assumed to be a volume detonation or an instantaneous detonation. The detonation front reaches the interface between the charge and the shell instantaneously and does not increase from the charge center to the inner surface of the shell. As a result, the detonation front in the theoretical model is inevitably lower than that in the experiment. This factor may account for the deviation in the velocity curves.

The second stage is between the disappearance of oscillation in experimental data and fragmentation of the natural cylindrical shell. In this stage, the shock wave that propagates back and forth in the shell gradually weakens, and the pressure of expanding gas begins to dominate the fragments acceleration [19]. As a result, the oscillation in experimental data disappears and the theoretical prediction is in good agreement with the experimental result. The gas pressure inside the shells decreases continuously due to expansion, thereby slowing down fragments acceleration. The final stage is after the rupture of the naturally fragmenting shell. The shells have now expanded to the place where the internal gas pressure insufficient to have a significant effect on the fragment acceleration. Thus, there is no evident velocity increase in the natural fragments.

Overall, despite the deviations in the early stage of shell expansion, the newly established model gives a more accurate estimation of the natural fragment acceleration than does Equation (3). In addition, the error between the theoretical prediction and the experimental data regarding the final fragment velocity is no more than 1.79% (see Table 1), indicating that the proposed formula is highly applicable to predict the post-disintegration acceleration of the naturally fragmenting shell.

### 3.2. Preformed Fragment Acceleration

To further validate the accuracy of the proposed model on the acceleration of preformed fragments, we conducted additional calculations and numerical simulations. The parameters for calculations and simulations are identical to those of the experiment (RD 9415) conducted by Predebon et al. [14].

In the experiment, the cubic preformed fragments fabricated from ANSI 1018 steel had a length of 7.95 mm. The high explosive was Octol with a density of 1.77 g/cm^3^ and a detonation velocity of 8200 m/s. The charge had a diameter of 126.04 mm and a length that was twice the diameter, and the ratio of charge mass to metal mass (*β*) was 0.926. The maximum velocity of fragments is around 1970 m/s, with a corresponding fragments separation radius of 1.298*R*_0_ [14,28].

Since the fragment acceleration is not captured by the flash radiographic observations in the experiment, we use numerical simulations with the ANSYS Autodyn software (ANSYS 15.0, ANSYS, Pittsburgh, PA, USA) for the detailed preformed fragment acceleration. The preformed-fragment simulation model has the same shell structure and *β* value as those in the experiment conducted by Predebon et al. [14].

#### 3.2.1. Numerical Simulation Model

**Figure 4:**
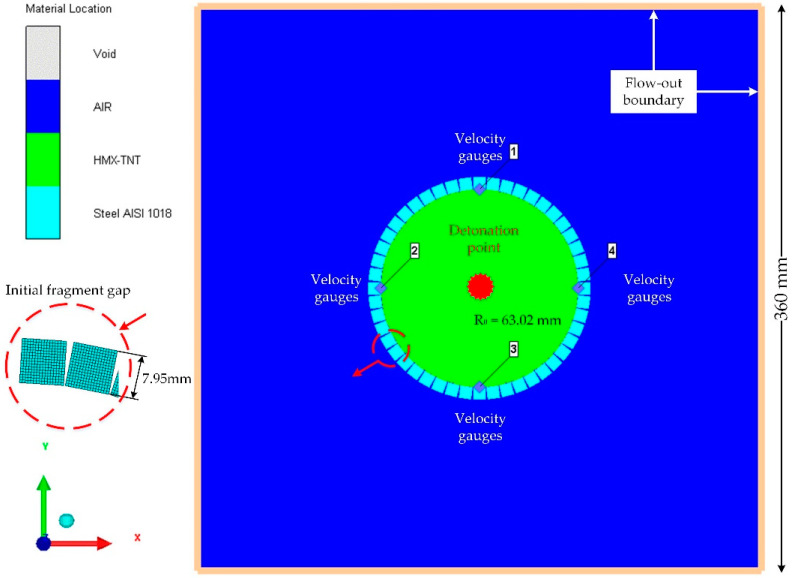
Geometries of the steel preformed-fragment cylinder used in the simulation.

The geometries of the steel preformed-fragment cylinder used for the simulation are shown in Figure 4. Since the recovered preformed fragments with the maximum velocity were free from the rarefaction wave from both ends, the simulation was simplified into a 2D plane model to compare with the established model. The fragments made of ANSI 1018 steel was modeled with Lagrange mesh, while the explosive charge was modeled with Euler mesh. ‘Flow out’ boundary conditions were applied to the air grid to avoid reflection of pressure. The dimensions of the air were around six times the inner radius of the charge, which is sufficient to achieve a stable velocity of fragments. We choose a mesh size of 0.5 mm for both the shell and the air as a compromise based on accuracy and computational efficiency.

For the air, we use the ideal gas equation, the parameter values for which are taken from the Autodyn library [29]. For the explosive, we use the standard Jones–Wilkins–Lee equation of state, namely

(29) $$P=C_{1}\left(1-\frac{\omega }{r_{1}\gamma }\right)e^{-r_{1}\gamma }+C_{2}\left(1-\frac{\omega }{r_{2}\gamma }\right)e^{-r_{2}\gamma }+\frac{\omega E}{\gamma }$$

where *P* is the detonation pressure, *E* is the internal energy per initial volume, and *V* is the initial relative volume. The other coefficients are material constants.

The Octol (a melt-castable, high explosive mixture consisting of HMX (cyclotetramethylenete-tranitramine) and TNT (trinitrotoluene)) used in the experiment was not a standard Octol explosive in the Autodyn material library and the material parameters of this non-ideal Octol were not available, therefore HMX-TNT explosive from the Autodyn material library [29] was selected as a replacement. The detonation speed and density of HMX-TNT are very close to those of the Octol explosive used in the experiment. The difference between the simulation results and the experimental data regarding the final fragment velocity is no more than 1.02% (see Table 1), indicating that the replacement is reasonable and the established numerical model can reliably predict the acceleration of preformed fragments. The parameter values for HMX-TNT are given in Table 2.

For the AISI 1018 steel, we use the Johnson–Cook model [30], in which the flow stress is expressed as

(30) $$\sigma =\left[A+B\varepsilon^{n}\right]\left[1+C\ln\varepsilon^{*}\right]\left[1-{\frac{\left(T-T_{room}\right)}{\left(T_{melt}-T_{room}\right)}}^{m}\right]$$

where ε is effective plastic strain, $\varepsilon^{*}$ is normalized equivalent plastic strain rate (typically normalized to a strain rate of 1.0 s^−1^), $T_{room}$ is room temperature, and $T_{melt}$ is the melting temperature of metal. *A* is initial yield stress, *B* is hardening constant, and *C* is strain rate constant. *n* is hardening exponent, and *m* is thermal softening exponent. The material properties used for the steel are listed in Table 3 [31].

**Figure 5:**
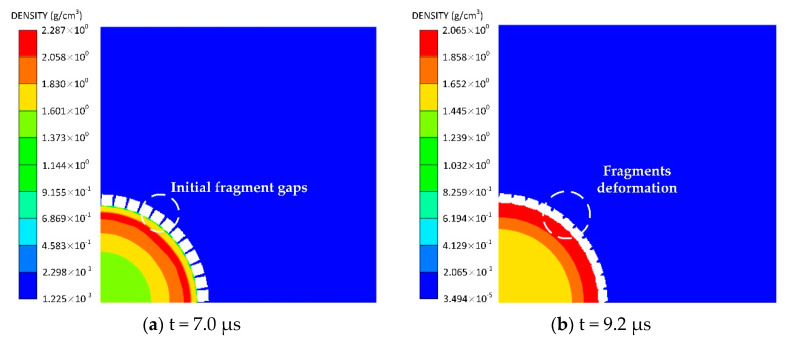

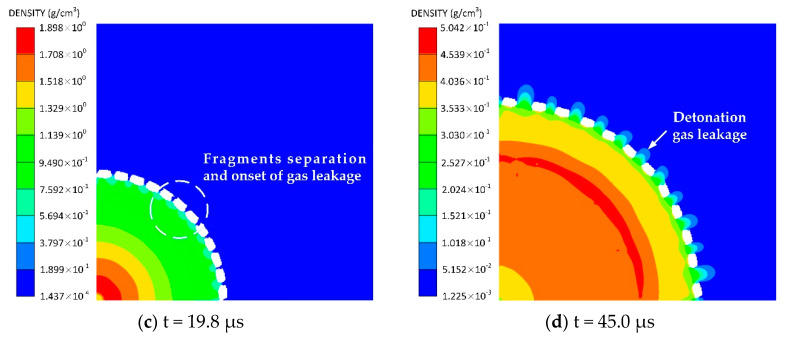
Density of Euler domain (detonation gas and air) after detonation of explosive: (**a**) t = 7.0 μs, (**b**) t = 9.2 μs, (**c**) t = 19.8 μs, (**d**) t = 45.0 μs. (**a**–**d**) shows the fragment deformation and separation process as well as the detonation gas leakage process.

To observe the fragment deformation and separation process as well as the detonation gas leakage process after the explosive is detonated, the density counter of the Euler domain (detonation gas and air) is shown in Figure 5. The high-density preformed fragments are shown as blank square blocks in the figure. Note that the presence of the initial fragment gaps is bound to cause a certain amount of gas outflow before the fragments gaps close. However, the Euler density counter shows no evident density change outside the preformed-fragment cylinder, indicating that the amount of leaked gas at this stage is negligible relative to the total amount of gas. After the shock wave propagates through the fragments, the fragments are observed to extend circumferentially to close the gaps. Upon reaching their maximum deformation, the fragments begin to separate under the drive of the wave reflected from the fragment free surface, whereupon the gas emerges from the gaps between the separated fragments. This finding is consistent with previous studies [24,28] that the internal detonation gas will not leak out until the preformed fragments separate. Therefore, the preformed-fragment cylinder can also be considered as a continuous cylinder, and the newly established theory can be utilized to calculate the acceleration of the separated fragments yielded by the preformed-fragment cylinder.

#### 3.2.2. Verification of Fragment Acceleration

Having conducted the simulation for the preformed-fragment cylinder, we turn to verify the theoretical model for detailed fragment acceleration and final fragment velocity. The critical parameter values for calculation are *R*_0_ = 63.02 mm, *β* = 0.926, *γ* = 3, *D* = 8200 m/s, *R_f_* = 81.80 mm *V_f_* = 1610 m/s, as shown in Table 1. As mentioned in Section 3.1, the detonation wave that initials from charge center and the detonation wave that initials from volume detonation may differ in pressure right at the interface between the charge and shell. Therefore, it is necessary to figure out the effect of detonation methods on the acceleration of the fragments. We added a new simulation with volume detonation to compare with the aforementioned simulation where the charge was initiated from the center and the proposed model. Since the problem can be simplified into a plane model, a plane detonation in Autodyn software (ANSYS 15.0, ANSYS, Pittsburgh, PA, USA) was selected to replace the assumed volume detonation. All the other parameters of simulation with plane detonation are identical to those of the simulation where the charge is initiated from the center.

**Figure 6:**
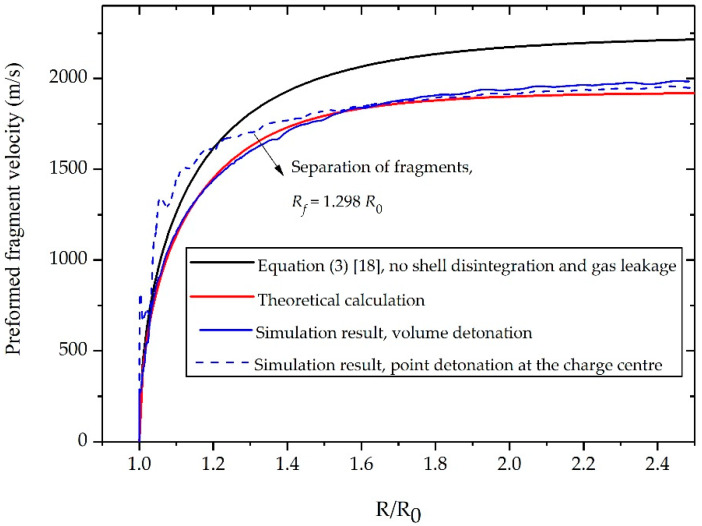
Velocity histories of fragments generated from a preformed-fragment fabricated from AISI 1018 steel.

The fragment velocity predicted by the present model along with the data from simulations and result calculated by Equation (3) are plotted in Figure 6. Our analysis shows that the simulation with volume detonation coincides better with the theoretical prediction than does the simulation with point detonation at the charge center especially in the early period of shell expansion. The deviations in the velocity curves can be explained by the fact that the detonation pressure at the interface between the gas and shell in the cases of point detonation is higher than that in the cases of volume detonation. However, the focus of this article is the post-separation acceleration of the discrete fragments. Despite the deviations in the early stage of shell expansion between the simulation with volume detonation and the simulation with point detonation, the differences between the two cases regarding the post-separation period and final fragment velocity is not evident, indicating that the established model with the assumption of volume detonation is also applicable to predict the acceleration of disintegrated shell where charge is initiated from the center. 

The comparison of the final fragment velocity among the proposed model, the experimental data, and other formulas is also presented in Table 1. It can be clearly seen that the established model gives a more accurate prediction compared to other formulas. The fragment velocity predicted by the proposed model coincides well with the experiment data, owing an error of 2.79%. This well coincidence indicates that the established model in the present work can reasonably predict the discrete fragments acceleration driven by explosives. 

## 4. Discussion

Although previous studies [16,18] have established the model for gas leakage and fragment acceleration, no data were found on the association between these critical factors. To facilitate a better understanding of the exact mechanism of the post-disintegration acceleration of a warhead shell, it is necessary to describe in greater detail the complicated interaction between gas leakage and fragment acceleration. Therefore, we take the preformed-fragment cylinder as an example, and we calculate all the variables iteratively as those in Section 3.2.

**Figure 7:**
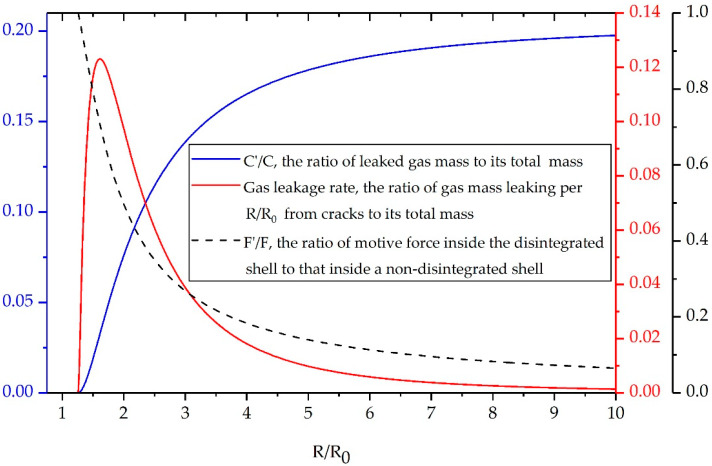
Gas mass loss (blue), leakage rate (red), and motive force drop (dashed black) versus *R*/*R*_0_.

Figure 7 shows the gas mass loss, the gas leakage rate, and the motive force drop inside disintegrated shells as functions of *R*/*R*_0_. At the shell disintegration radius, the high-pressure gas flows out through the cracks because of the relatively low pressure outside the casing, whereupon fragments gaps extension increases the gas leakage rate and gas mass loss. Meanwhile, the inner surface area of the shell ceases to increase after disintegration and the gas pressure decreases due to gas leakage, thereby leading to a sharp drop in the motive force that accelerates fragments outwards. However, as the shell expands continuously, the gas leakage rate reaches a maximum and then decreases continuously. In addition, the gas mass loss and motive force changes more slowly and eventually stabilize in the late period of shell expansion. This tendency may be due to the competition between the gas leakage and fragments acceleration. On the one hand, the leaked gas reduces the internal gas pressure, and this may prevent the fragments and remaining gas and from obtaining sufficient motive force to accelerate outwards. On the other hand, the increase in fragment velocity causes the gas to escape at a relatively low velocity and further decreases the motive force due to shell expansion. Finally, the motive force will drop to a value that is insufficient to maintain a high leakage rate and to accelerate the disintegrated shell, and the leakage will stop when the outflow of gas keeps pace with the expansion of the shell. 

One interesting finding is that the final gas mass loss of the preformed-fragment cylinder is 20.2% of the initial charge mass, which is consistent with the work of Charron [24] who reduced 20% initial charge mass to account for the effect of gas leakage on fragment acceleration. However, the gas mass loss is only 13% when the fragments reach a stable velocity at three times *R*_0_. Several factors could explain this observation. Firstly, the increase in fragment velocity competes with the gas leakage, which slows down the gas leakage compared with that predicted by Charron. Secondly, the shell disintegration and gas leakage will cause a combined hinder effect on the fragment acceleration. In other words, the 13% leaked gas in our model can result in a greater fragment velocity loss compared to that in Charron’s model. Thirdly, after the separated preformed-fragment cylinder expands to three times *R*_0_, the motive force inside the shell has dropped to a value which is not sufficient to accelerate fragments due to the shell expansion and gas leakage. As a result, though the internal gas continuously flows out through the fragment gaps, the leaked gas will affect little on fragment acceleration and can be ignored. These encouraging findings, though not intuitive, may provide an insight into the essence of the acceleration characteristics of discrete fragments driven by explosives.

## 5. Conclusions

Considering the combined effect of shell disintegration and gas leakage, we established a theoretical model to describe the post-disintegration acceleration of a cylindrical metallic shell driven by explosives, based on the modification of the motive force on discrete fragments by fragment-area change after the shell breaks up and subsequent gas-pressure drop due to gas leakage. The theoretical calculation results agreed well with experimental data and numerical simulations.

The gas leakage was found to compete with the acceleration of the disintegrated shell. On the one hand, as the cracks expand, the gas leakage rate increases, and the leakage reduces the gas pressure and motive force inside the shell significantly, resulting in a lower fragment velocity compared with that for an intact shell. On the other hand, the increase in fragment velocity slows down the gas leakage rate and further decreases the motive force that pushes fragments and gas products outwards. This complicated interaction will stop when the gas outflow keeps pace with the expansion of the discrete fragments. 

Note that the state quantities inside an expanding shell, such as gas pressure and motive force, are challenging to measure and verify experimentally. In addition, due to the assumption of instantaneous detonation, the formula may not predict the acceleration of the warhead shell accurately in the initial expansion period. Another limitation of this study is that we do not pay much attention to the physical mechanisms of shell deformation and subsequent fragmentation. In fact, the metallic shell is driven to expand rapidly by the high-intense shock, followed by large plastic or thermoplastic deformations that ultimately lead to rupture. Physical mechanisms of shell deformation and fracture information are important aspects of dynamic response of cylindrical shells driven by explosives and should receive considerable attention. However, in this article, we do not go into the details of the shell deformation and the crack propagation and we use the deformation and critical fracture information from the reported literatures. We wish to extend this topic in our continued study.

Despite these limitations, the present study gives a clear and accurate theoretical prediction of the post-disintegration acceleration of a warhead shell. The velocity distribution in the axial direction of the warhead may differ from that in the radial direction under the combined effect of the rarefaction wave and gas leakage. Therefore, we plan to extend the present study by incorporating the end effects of warheads with gas leakage to establish a model that can predict the fragment velocity in the axial direction of a cylindrical warhead. 

## Figures and Tables

**Table 1:** The critical parameter values for theoretical calculations and results of calculations on final fragment velocity.

**Numbers**	**Fragment Type**	**The Critical Parameter Values for Calculation**						
		***R*_0_ (m)**	***R_f_* (m)**	***β***	***γ***	***D* (m/s)**	***V_f_* (m/s) ^1^**	**Step**
1-1	Natural fragment	0.025	0.04575	0.4	3	6700	1167	1% *R*/*R*_0_
1-2	Preformed fragment	0.06302	0.0818	0.926	3	8200	1610	1% *R*/*R*_0_
**Numbers**	**Fragment Type**	**Final Fragment Velocity (m/s)**						
		**Gurney Formula [1]**	**Theoretical Prediction**	**Experimental Data [4,14]**	**Charron Formula [24]**	**Kim Formula [25]**	**Numerical Simulation**	**Error**
1-1	Natural fragment	1330	1209	1231 [4]	—	—	—	1.79%
1-2	Preformed fragment	2243	1915	1970 [14]	2073	1810	1990 ^2^, 2000 ^3^	2.79%

^1^*V_f_* refers to the velocity when the shell fractures into discrete fragments. ^2^ Error of simulation where the charge is initiated from the center compared to experimental data is 1.02%. ^3^ Error of simulation with a volume detonation compared to experimental data is 1.52%.

**Table 2:** Parameter values for HMX(cyclotetramethylenete-tranitramine)-TNT(trinitrotoluene) explosive [29].

Density (g/cm^3^)	Detonation Velocity (*D*, m/s)	C-J Pressure (GPa)	*E*_0_ (kJ/m^3^)	*C*_1_ (GPa)	*C*_2_ (GPa)	*r*_1_	*r*_2_	*ω*
1.776	8210	31.1	8.9 × 10^6^	700	12.12	4.5	1.1	0.3

**Table 3:** Parameters of AISI 1018 steel [31].

Material	Density (g/cm^3^)	*A* (MPa)	*B* (MPa)	*n*	*C*	*m*	*T_melt_* (K)
1018 steel	7.9	735	309	0.44	0.0064	1.05	1793
